# Sonographic characteristics and clinical characteristics combined with nomogram for predicting the aggressiveness of papillary thyroid carcinoma coexisted with Hashimoto’s thyroiditis

**DOI:** 10.1016/j.bjorl.2024.101456

**Published:** 2024-06-10

**Authors:** Shuangshuang Zhao, Zheng Zhang, Xin Zhang, Xincai Wu, Yanwei Chen, Xin Min, Baoding Chen

**Affiliations:** Affiliated Hospital of Jiangsu University, Department of Ultrasound Medicine, Zhenjiang, Jiangsu Province, China

**Keywords:** Papillary thyroid carcinoma, Hashimoto’s thyroiditis, Aggressiveness, Ultrasonography, Nomogram

## Abstract

**Objective:**

The association between Papillary Thyroid Carcinoma (PTC) and coexistent Hashimoto’s Thyroiditis (HT) was controversial. The purpose of this study was to evaluate the presence of HT exerts any influence on the aggressiveness of PTC, and to establish a nomogram for predicting the possibility of aggressiveness in PTC.

**Methods:**

373 consecutive PTC patients with/without coexistent HT from January 2017 to December 2020 were retrospective reviewed. Patients’ clinicopathologic and sonographic characteristics were collected for univariate and multivariate analyses. A nomogram was established based on the risk factors for aggressiveness in PTC.

**Results:**

Male (*p* = 0.001), tumor size >1.0 cm (*p* = 0.046) and lymph node metastasis (*p* = 0.018) were negatively associated with PTC coexisted with HT, while it was significantly positively associated with the frequence of multifocality (*p* = 0.010). Univariate and multivariate analyses suggested that age ≥55 years (*p* = 0.000), male (*p* = 0.027), HT (*p* = 0.017), tumor size >1.0 cm (*p* = 0.015), multifocality (*p* = 0.041), distance to capsular ≤0 cm (*p* = 0.050) and blood flow (Grade I: *p* = 0.044) were independent risk factors for predicting the aggressiveness in PTC. A nomogram according to these predictors was further developed and validated. The receiver operating characteristic curve (AUC = 0.734 and 0.809 for training and validation cohorts, respectively) and decision curve analyses indicated that the nomogram model was clinically useful. The calibration curve revealed that the nomogram exhibited an excellent consistency.

**Conclusions:**

In this study, the coexistent HT might play a protective role in preventing the proliferation of PTC. Dispensable aggressive treatment may be reduced in PTC by pre-operative identification of sonographic and clinical characteristics and incorporating with the predicted nomogram model.

## Highlights

• HT played a protective role in PTC.
• Blood flow was a risk factor of aggressiveness in PTC.
• A nomogram for predicting the aggressiveness of PTC.

## Introduction

Evidence level: This article’s evidence level is 3. Level 3 evidence is derived from non-randomized, controlled clinical trials. In this study, patients who receive an intervention are compared to a control group. Authors may detect a statistically significant and clinically relevant outcome.

Papillary Thyroid Carcinoma (PTC) accounts for the highest proportion (more than 90%) of all thyroid carcinomas.^1^ Most PTCs have an indolent disease course with a low mortality rate and a favorable prognosis even after low-intensity treatment.^2^ In consequence, a wide range of options are employed for disease management, from active surveillance to surgical treatment and subsequent radioactive iodine ablation.^3^ Hence the stratification of PTC patients by risk of aggressiveness has been the main clinical issue. Several clinicopathologic characteristics have been currently recognized as risk factors of unfavorable prognosis, such as older age, large primary tumor size, Extrathyroidal Extension (ETE), Lymph Node Metastasis (LNM), and Distant Metastasis (DM).^4^ According to ATA guideline, aggressive treatment is recommended for PTC patients with these risk factors. Otherwise, low-intensity treatment may be sufficient.^3^ Nevertheless, these managements are all based on clinicopathologic analysis, resulting in excessive unnecessary surgeries. Preoperative multifaceted accurate evaluation must be carried out by identification of specific biomarkers, invasive sonographic features and aggressive clinical characteristics.^5^

Hashimoto’s Thyroiditis (HT) occupies the major proportion of autoimmune thyroid disorders, causing chronic inflammation of the thyroid tissue.^5^ The coexistence rate of PTC combined with HT reported in epidemiologic studies is increasing, ranging from 5% to 85%.^6^, ^7^ Although the existence of association between HT and PTC development and progression has been accepted, there always been controversial. Due to the less invasive disease and the lower recurrence rate, coexistent HT is widely believed playing a protective role in PTC.^8^ Lymphocyte infiltration caused by HT and facilitating antitumor immunity maybe connected with this function, which makes the association rational.^9^ However, few studies regarding the association of HT with PTC in aggressiveness by sonographic characteristics of PTC with the presence of coexistent HT. This study was aimed at assessing and determining the clinical characteristics and sonographic characteristics for predicting the aggressiveness of PTC coexisted with HT, by univariate analysis, multivariate analysis and nomogram.

## Methods

### Patients

**Figure 1:**
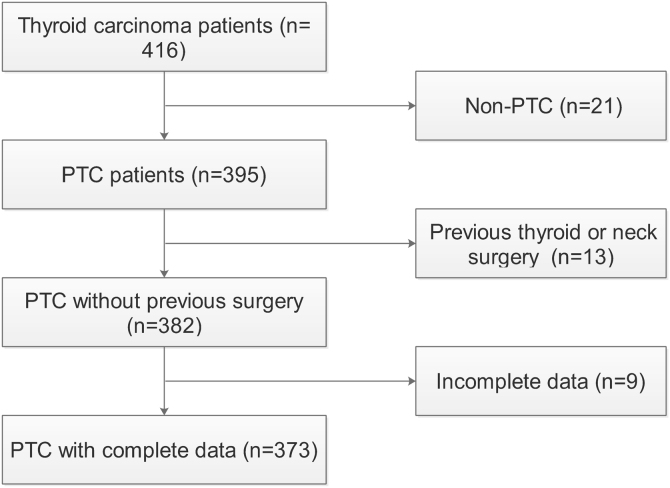
Flow chart of this study. PTC, Papillary Thyroid Carcinoma.

This study was conducted according to Declaration of Helsinki and approved by the Institutional Ethical Committee. Three hundred and seventy-three consecutive patients (275 females and 98 males; age range: 18–80 y; mean age: 45.04 ± 12.27 y) were enrolled from January 1^st^, 2017 to December 31^st^, 2020 in this study (Fig. 1). In addition, we collected 111 patients (86 females and 25 males; age range: 21–68 y; mean age: 42.76 ± 11.24 y) as validation cohort from January 1^st^ to August 31^st^, 2021. All patients received lobectomy or total thyroidectomy for PTC with/without coexistent HT at our hospital. Informed consent forms for general use of clinical information in future studies were obtained at the time of operation.

Inclusion criteria were (1) Patients ≥18 years old with thyroid nodules (Bethesda system ≥ V cytology). Exclusion criteria were (1) Patients with incomplete documents; (2) Patients underwent surgery for poorly differentiated thyroid cancer or other thyroid malignant neoplasms; (3) Patients with history of cancer or thyroid surgery. For patients who had standard indications, therapeutic neck dissection was performed.

### Image analysis

**Figure 2:**
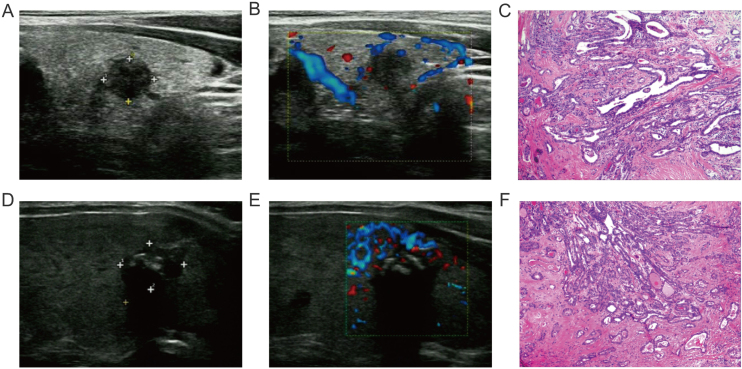
(A‒C) Conventional ultrasonography, CDFI and pathological tissue image of PTC without coexistent Hashimoto’s thyroiditis. (D‒F) Conventional ultrasonography, CDFI and pathological tissue image of PTC with coexistent Hashimoto’s thyroiditis.

All patients were detected by conventional Ultrasound (US) and color Doppler US. Sonographic characteristics include tumor size >1.0 cm, marked hypoechoic, taller than wide, distance to capsular ≤0 cm, microcalcification, irregular margin and multifocality (Fig. 2A‒B, D‒E). Blood flow of nodules was classified into the following 3 levels: Grade 0, no blood flow in the nodule; Grade I, the nodules show a small amount of blood flow, only a few spots of blood flow or one long vessel penetrating into the nodule (more than half of the maximum diameter of the nodule); and Grade II, there is abundant blood flow inside the nodule, with 5 or more punctate blood flow or 2 long vessels.^10^ These sonographic characteristics were all confirmed by two experienced US experts using MyLab Twice (Esaote, Italy) with the probe LA523.

### Histopathology

Standard pathologic diagnosis followed the World Health Organization criteria. Coexistent HT, primary tumor size, ETE, gross ETE, and LNM of thyroid were confirmed by two pathologists with at least 5 years of working experience (Fig. 2C‒F). HT was diagnosed on pathological examination of surgical specimen to avoid the confounding effects of autoimmune antibodies.^11^ Absent LNM was defined, if one patient who did not undergo lateral compartment dissection with his negative result of US examination for lymph nodes. In this study, PTC was defined as aggressiveness, when ETE, gross ETE, or LNM was showed in pathological histology result.

### Statistical analysis

Mean ± Standard Deviation (SD) indicated quantitative data. Student’s *t*-test, Pearson χ^2^ test or Fisher’s exact test were used for clinicopathologic characteristics and sonographic characteristics compared across groups. Multivariate logistic regression analysis was used in order to identify risk factors for PTC with/without coexistent HT and to identify risk factors for aggressiveness in PTC. The nomogram was established for predicting the aggressiveness of PTC in patients. The diagnostic accuracy of prediction of aggressiveness was calculated with Receiver Operating Characteristic (ROC) analysis. The discriminative ability of the predictive nomogram was assessed by Harrell’s Concordance Index (C-index). The calibration curve was used to determine the prediction compliance. The Decision Curve Analysis (DCA) was used to evaluate the clinical application value of the model; *p-*value < 0.05 was considered statistically significant. All statistical analyses were performed using the SPSS 26.0 statistical package (SPSS, Inc., Chicago, IL, USA), and R language software and R Studio.

## Results

A total of 373 patients were incorporated into study, including 275 women (73.7%). The prevalence of PTC with coexistent HT was 20.4% (76 cases). The average age of patient with PTC absent of HT was 44.98 ± 12.28 years, and the average age of PTC patient coexistent with HT was 45.30 ± 12.30 years.

Female occupied a major component of patients with coexistent HT compared to patients absent of HT (90.8 % vs. 69.4 %). Compared with PTC absent of HT, PTC with coexistent HT had a smaller tumor size (0.85 ± 0.49 cm vs. 1.07 ± 0.73 cm) and were less likely to have ETE (1.3% vs. 8.1%) and LNM (42.1% vs. 59.9%) (all *p* <  0.05). Oppositely, PTC with coexistent HT was more likely to have multifocality (39.5% vs. 23.6%, *p* <  0.05). Nodule size >1.0 cm was the only sonographic characteristics between PTC with coexistent HT group and the group without coexistent HT (21.1% vs. 35.0%, *p* < 0.05). There was no significance between the two groups for the other sonographic characteristics of PTC for univariate analysis (Table 1). The association of clinical characteristics and sonographic characteristics between the PTC patients with and without coexistent HT was assessed using multivariable analysis including gender, tumor size, ETE, LNM and multifocality. Table 1 shows that male (OR = 0.233; 95% CI 0.100‒0.540; *p* = 0.001), tumor size >1.0 cm (OR = 0.512; 95% CI 0.265‒0.988; *p* = 0.046) and LNM (OR = 0.491; 95% CI 0.272‒0.885; *p* = 0.018) were significantly negatively associated with frequencies of PTC with coexistent HT. Nevertheless, multifocality (OR = 2.138; 95% CI 1.195‒3.825; *p* = 0.010) was positively associated with PTC with coexistent HT.

**Table 1:** Univariate and multivariate analysis of clinical, pathological and sonographic characteristics in PTC patients.

Variables	Univariate analysis			Multivariate analysis		
	HT absent (n = 297)	HT Present (n = 76)	*p*-value	OR	95% CI	*p*-value
Age ≥55 years, n (%)			0.133			
No	228 (76.8)	52 (68.4)				
Yes	69 (23.2)	24 (31.6)				
Gender, n (%)			0.000	0.233	0.100‒0.540	0.001
Female	206 (69.4)	69 (90.8)				
Male	91 (30.6)	7 (9.2)				
Tumor size >1.0 cm, n (%)			0.020	0.512	0.265‒0.988	0.046
No	193 (65.0)	60 (78.9)				
Yes	104 (35.0)	16 (21.1)				
ETE, n (%)			0.035	0.161	0.020‒1.313	0.088
No	273 (91.9)	75 (98.7)				
Yes	24 (8.1)	1 (1.3)				
Gross ETE, n (%)			0.508			
No	266 (89.6)	70 (92.1)				
Yes	31 (10.4)	6 (7.9)				
Multifocality, n (%)			0.005	2.138	1.195‒3.825	0.010
No	227 (76.4)	46 (60.5)				
Yes	70 (23.6)	30 (39.5)				
Bilateral lesions, n (%)			0.407			
No	243 (81.8)	59 (77.6)				
Yes	54 (18.2)	17(22.4)				
LNM, n (%)			0.002			0.013
None	119 (40.1)	44 (57.9)				
N1a	160 (53.9)	24 (31.6)		0.491	0.272‒0.885	0.018
N1b	18 (6.0)	8 (10.5)		1.696	0.591‒4.867	0.326
Marked hypoechoic, n (%)			0.562			
No	76 (25.6)	17 (22.4)				
Yes	221 (74.4)	59 (77.6)				
Taller than wide, n (%)			0.194			
No	163 (54.9)	48 (63.2)				
Yes	134 (45.1)	28 (36.8)				
Distance to capsular ≤0 cm, n (%)			0.217			
No	201 (67.7)	57 (75.0)				
Yes	96 (32.3)	19 (25.0)				
Microcalcification, n (%)			0.398			
No	61 (20.5)	19 (25.0)				
Yes	236 (79.5)	57 (75.0)				
Irregular Margin, n (%)			0.241			
No	65 (21.9)	12 (15.8)				
Yes	232 (78.1)	64 (84.2)				
Blood flow, n (%)			0.529			
Grade 0	86 (29.0)	19 (25.0)				
Grade I	112 (37.7)	34 (44.7)				
Grade II	99 (33.3)	23 (30.3)				

HT, Hashimoto Thyroiditis; ETE, Extrathyroidal Extension; LNM, Lymph Node Metastasis.

Results of univariate analysis and multivariate analysis for aggressive risk factors of PTC are shown in Table 2. Clinical (age ≥55 years; gender; HT) and sonographic characteristics (tumor size >1.0 cm; multifocality; distance to capsular ≤0 cm; microcalcification; blood flow) were significantly different between aggressive group and absent group (all *p* < 0.05). Significant results were subjected to multivariate logistic regression analysis. Age ≥55 years (OR = 0.359; 95% CI 0.213‒0.604; *p* = 0.000), male (OR = 1.871; 95% CI 1.075‒3.254; *p* = 0.027), HT (OR = 0.496; 95% CI 0.279‒0.882; *p* = 0.017), tumor size >1.0 cm (OR = 2.031; 95% CI 1.146‒3.600; *p* = 0.015), multifocality (OR = 1.770; 95% CI 1.023‒3.063; *p* = 0.041), distance to capsular ≤0 cm (OR = 1.750; 95% CI 1.001‒3.061; *p* = 0.050) and blood flow (Grade I: OR = 1.788; 95% CI 1.015‒3.149; *p* = 0.044; Grade II: OR = 1.741; 95% CI 0.912‒3.321; *p* = 0.093) were risk factors for aggressiveness in PTC.

**Table 2:** Univariate and multivariate analysis of aggressive characteristics in PTC patients.

Variables	Univariate analysis			Multivariate analysis		
	Absent (n = 151)	Aggressive (n = 222)	*p*-value	OR	95% CI	*p-*value
Age ≥55 years, n (%)			0.000	0.359	0.213‒0.604	0.000
No	96 (63.6)	184 (82.9)				
Yes	55 (36.4)	38 (17.1)				
Gender, n (%)			0.020	1.871	1.075‒3.254	0.027
Female	121 (80.1)	154 (69.4)				
Male	30 (19.9)	68 (30.6)				
HT			0.001	0.496	0.279‒0.882	0.017
No	108 (71.5)	189 (85.1)				
Yes	43 (28.5)	33 (14.9)				
Tumor size >1.0 cm, n (%)			0.000	2.031	1.146‒3.600	0.015
No	122 (80.8)	131 (59.0)				
Yes	29 (19.2)	91 (41.0)				
Multifocality, n (%)			0.043	1.770	1.023‒3.063	0.041
No	119 (78.8)	154 (69.4)				
Yes	32 (21.2)	68 (30.6)				
Bilateral lesions, n (%)			0.070			
No	129 (85.4)	173 (77.9)				
Yes	22 (14.6)	49 (22.1)				
Marked hypoechoic, n (%)			0.566			
No	40 (26.5)	53 (23.9)				
Yes	111 (73.5)	169 (76.1)				
Taller than wide, n (%)			0.763			
No	84 (55.6)	127 (57.2)				
Yes	67 (44.4)	95 (42.8)				
Distance to capsular ≤0 cm, n (%)			0.000	1.750	1.001‒3.061	0.050
No	121 (80.1)	137 (61.7)				
Yes	30 (19.9)	85 (38.3)				
Microcalcification, n (%)			0.000	1.608	0.910‒2.841	0.102
No	46 (30.5)	34 (15.3)				
Yes	105 (69.5)	188 (84.7)				
Irregular Margin, n (%)			0.129			
No	37 (24.5)	40 (18.0)				
Yes	114 (75.5)	182 (82.0)				
Blood flow, n (%)			0.000			0.104
Grade 0	59 (39.1)	46 (20.7)				
Grade I	53 (35.1)	93 (41.9)		1.788	1.015‒3.149	0.044
Grade II	39 (25.8)	83 (37.4)		1.741	0.912‒3.321	0.093

HT, Hashimoto Thyroiditis.

**Figure 3:**
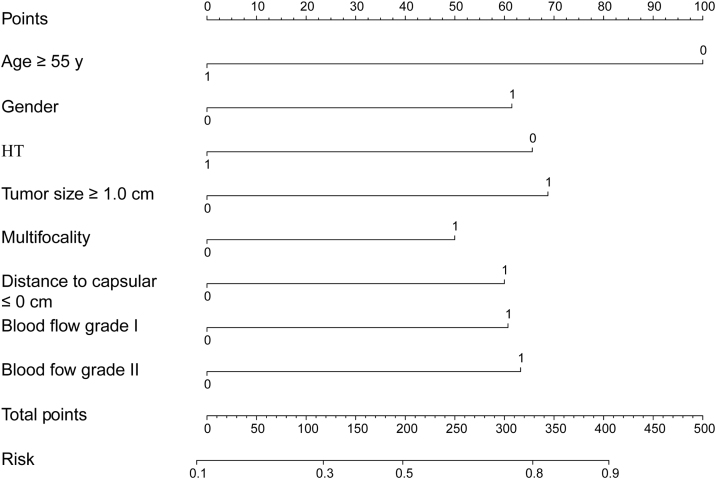
Nomogram for predicting the aggressiveness of PTC in patients.

**Figure 4:**
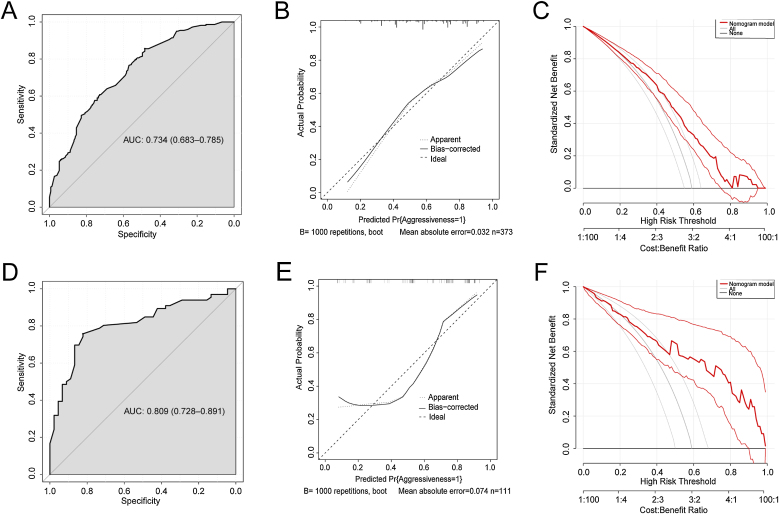
(A) ROC curve analysis of training cohort for predicting the aggressiveness in PTC patients. (B) Calibration plots of training cohort for predicting the aggressiveness of PTC in patients. (C) DCA of training cohort for predicting the aggressiveness of PTC in patient. (D) ROC curve analysis of validation cohort for predicting the aggressiveness in PTC patients. (E) Calibration plots of validation cohort for predicting the aggressiveness of PTC in patients. (F) DCA of validation cohort for predicting the aggressiveness of PTC in patient.

A relevant predictive nomogram was established with integrated clinical (age ≥55 years; gender; HT) and sonographic factors (tumor size >1.0 cm; multifocality; distance to capsular ≤0 cm; blood flow) to assist in preoperative predicting aggressiveness of PTC (Fig. 3). The nomogram each level within variables was assigned a score according to the point scale. By adding the total score and locating it on the total point scale, a corresponding probability of aggressiveness of each individual was determined. No significant differences were noted in clinical and sonographic characteristics between the training cohort and validation cohort (Table 3). AUC value of ROC curve was 0.734 (0.683‒0.785) in the training cohort and was 0.809 (0.728‒0.891) in the validation cohort (Fig. 4A and D). The C-index of this nomogram was 0.734, indicating that the nomogram model had good, predicted accuracy. The calibration curves of training cohort and validation cohort revealed that the nomogram exhibited an excellent consistency (Fig. 4B and E). The DCA demonstrated that predicting aggressiveness applying this model would be better than having all patients or none patients with a range of the threshold probability ranged from 0.2 to 0.8 in training cohort, and from 0.2 to 1.0 in validation cohort (Fig. 4C, F).

**Table 3:** Clinical, pathological and sonographic characteristics in training cohort and validation cohort.

Variables	Patients (n = 484)		*p*-value
	Training (n = 373)	Validation (n = 111)	
Age ≥55 years, n (%)			0.087
No	280 (75.1)	92 (82.9)	
Yes	93 (24.9)	19 (17.1)	
Gender, n (%)			0.426
Female	275 (73.7)	86 (77.5)	
Male	98 (26.3)	25 (22.5)	
Tumor size >1.0 cm, n (%)			0.267
No	253 (67.8)	69 (62.2)	
Yes	120 (32.2)	42 (37.8)	
ETE, n (%)			0.085
No	348 (93.3)	98 (88.3)	
Yes	25 (6.7)	13 (11.7)	
Gross ETE, n (%)			0.075
No	336 (90.1)	106 (95.5)	
Yes	37 (9.9)	5 (4.5)	
Multifocality, n (%)			0.196
No	273 (73.2)	88 (79.3)	
Yes	100 (26.8)	23 (20.7)	
Bilateral lesions, n (%)			0.074
No	302 (81.0)	98 (88.3)	
Yes	71 (19.0)	13 (11.7)	
LNM, n (%)			0.642
None	163 (43.7)	49 (44.1)	
N1a	184 (49.3)	57 (51.4)	
N1b	26 (7.0)	5 (4.5)	
Marked hypoechoic, n (%)			0.053
No	93 (24.9)	38 (34.2)	
Yes	280 (75.1)	73 (65.8)	
Taller than wide, n (%)			0.422
No	211 (56.6)	58 (52.3)	
Yes	162 (43.4)	53 (47.7)	
Distance to capsular ≤0 cm, n (%)			0.083
No	258 (69.2)	67 (60.4)	
Yes	115 (30.8)	44 (39.6)	
Microcalcification, n (%)			0.809
No	80 (21.4)	25 (22.5)	
Yes	293 (78.6)	86 (77.5)	
Irregular Margin, n (%)			0.057
No	77 (20.6)	14 (12.6)	
Yes	296 (79.4)	97 (87.4)	
Blood flow, n (%)			0.471
Grade 0	105 (28.2)	25 (22.5)	
Grade I	146 (39.1)	45 (40.5)	
Grade II	122 (32.7)	41 (36.9)	
HT			0.736
No	297 (79.6)	90 (81.1)	
Yes	76 (20.4)	21 (18.9)	
Aggressiveness			0.991
No	151 (40.5)	45 (40.5)	
Yes	222 (59.5)	66 (59.5)	

ETE, Extrathyroidal Extension; LNM, Lymph Node Metastasis; HT, Hashimoto Thyroiditis.

## Discussion

Although most PTC accompanied with an indolent clinical course in patient’s life, it is of great significance that a few PTC possess high risk of tumor invasion and metastasis, and even patient mortality.^12^ HT was firstly described as autoimmune thyroiditis with the most significant signs of atrophy of follicular cells, lymphocytic infiltration, goiter and fibrosis.^13^ As the most common autoimmune disease, HT is the most frequently diagnosed concomitant disease in patients with PTC.^8^ Some study has illustrated the vital relationship between coexistent HT and the less aggressive clinicopathologic characteristics in PTC, but the association about prognosis remains controversial. A cohort study suggested coexistent HT with PTC had a significant negative connection with PTC-related mortality.^14^ Whether the relationship between decrease of aggressiveness in PTC with coexistent HT is such of causal relationship remains to be illustrate. At present, pathologic characteristics like large tumor size, ETE, LNM, and DM have been considered as risk factors, maybe an increased risk of contralateral malignancy.^15^ Patients with these unfavorable factors require aggressive treatment, otherwise low-intensity treatment may be sufficient. In this study, we determined that PTC was aggressive based on the positive pathological findings with ETE, gross ETE, or LNM, and found that age <55 years, male, the presence of HT, tumor size >1.0 cm, multifocality, distance to capsular ≤0 cm and blood flow (Grade I and Grade II) were independent risk predictors for aggressive characteristics based on the multivariate logistic regression analysis.

The Central Neck Lymph Node (CLNM) status is important for the treatment strategy of PTC, thus lots of studies aimed to diagnose LNM preoperatively. A retrospective and cross-sectional study with 4131 PTC patients considered HT as a protective factor for both CLNM and Lateral Lymph Node Metastasis (LLNM) in PTC.^16^ A series of prognostic studies with sufficiently long follow-up suggested that more favorable outcome for PTC when coexistent HT was detected.^14^ Similar results were obtained in our study, PTC with coexistent TH was negatively connected with LNM (N1a) (*p* = 0.018). In other words, coexistent HT had a protective effect on CLNM. Grossly apparent invasion of thyroid cancer beyond the thyroid gland has been recognized an adverse feature for decades because of the connection with disease recurrence and death.^17^ The prevalence of an ETE was significantly lower in patients with PTC coexisted with HT in a Meta-analysis.^2^ Our result was similar. However, there was no association between HT and ETE in this study.

Previous studies demonstrated that the morbidity of PTC is relatively higher in women, while the rates of malignancies and mortality are higher in men.^18^, ^19^ Our study showed similar results, that PTC had a lower incidence in male than in female either with coexistent HT or not, and the male sex had a significantly positive association with the aggressiveness of PTC with coexistent HT (OR = 39.453; 95% CI 1.753‒887.898; *p* = 0.021). This may be related to the higher incidence of HT in female than in male.^18^ To some degree, a lower rate of HT in men may suggest more aggressive behavior and possibly a worse prognosis.^20^

Sonographic characteristics like tumor size >1.0 cm and multifocality in this study were considered had role in of PTC coexisted HT. PTC with coexistent HT had a smaller tumor size (0.85 ± 0.49 cm) than that PTC absent of HT (1.07 ± 0.73 cm) (*p* = 0.003), and it was negatively associated with tumor size >1.0 cm in this study. Concordant with our data, Zhou et al. demonstrated that the presence of HT may have a significant protective role in reducing tumor volume, alleviating capsule infiltration and lowering the chance for more advanced stages of differentiated thyroid cancer. In addition, the presence of HT in advanced may limit the tumor growth to the primary site.^21^ In contrast, Baser et al. reported that HT did not affect sonographic characteristics in patients with PTC. It is worth noting that the sonographic characteristics we selected out (tumor size >1.0 cm and multifocality) were not considered in that study.^22^ Numerous studies demonstrated that the rate of multifocality is higher in PTC patients with coexistent HT.^2^, ^23^, ^24^ Patients with multifocal PTC are at increased risk for LNM, DM, local recurrence after initial treatment, and regional recurrence.^24^, ^25^ Together, these observations suggest that aggressive sonographic characteristics might predict a poor prognosis, and aggressive treatment should be considered with these vital factors. Blood flow, a common sonographic characteristic, did not shown any relation to PTC with coexistent HT in previous studies. Nevertheless, our study revealed that blood flow had an important correlation with the aggressiveness of PTC for the first time. The blood flow classified into Grade I or Grade II were considered as risk factors in the nomogram model, the total score would be higher, and the corresponding probability of aggressiveness of each individual would be higher either.

This study has some limitations. First, it was a retrospective study, and therefore, selection bias was unavoidable. Second, absent LNM was considered in the light of negative result of ultrasound examination without histopathological results in the present study. Third, DM, the aggressive characteristic was not considered in this study for there was no distant metastasis in these patients until the retrospective study finished.

## Conclusions

In conclusion, the coexistent HT might play a protective role in preventing the proliferation of PTC. Dispensable aggressive treatment may be reduced in PTC patients especially coexisted with HT by pre-operative identification of sonographic (HT, tumor size >1.0 cm, multifocality, distance to capsular ≤0 cm and blood flow (Grade I and Grade II) and clinical characteristics (age ≥55 years and gender) and incorporating with the predicted nomogram model.

## Funding

This work was supported by the National Science Foundation for Young Scientists of China (grant number 82302208); the Medical research project of Jiangsu Provincial Health Commission (grant number H2023141); the Social Development Program of Zhenjiang City (grant numbers SH2022066, SH2023015, SH2023019); the Sixth Phase “169 Project” Scientific Research Project of Zhenjiang City (grant number YLJ202104); and the Medical Education Collaborative Innovation Fund of Jiangsu University (grant number JDYY2023012).

## Conflicts of interest

The authors declare no conflicts of interest.
